# Association between MKRN3 and LIN28B polymorphisms and precocious puberty

**DOI:** 10.1186/s12863-018-0658-z

**Published:** 2018-07-27

**Authors:** Bo Ram Yi, Hyun Jeong Kim, Hye Sook Park, Yoon Jeong Cho, Ju Young Kim, Jeong Yee, Jee Eun Chung, Joo Hee Kim, Kyung Eun Lee, Hye Sun Gwak

**Affiliations:** 1 College of Pharmacy, Chungbuk National University, 660-1, Yeonje-ri, Osong-eup, Heungdeok-gu, Cheongju-si, 28160 Republic of Korea; 2 Department of Preventive Medicine, Ewha Womans University, College of Medicine, Seoul, 07985 South Korea; 3 College of Pharmacy & Division of Life and Pharmaceutical Sciences, Ewha Womans University, 52 Ewhayeodae-gil, Seodaemun-Gu, Seoul, 03760 South Korea; 4 College of Pharmacy, Hanyang University, Ansan, 15588 South Korea; 5 College of Pharmacy, Ajou University, Suwon, 16499 South Korea

**Keywords:** Precocious puberty, Makorin RING finger protein 3, Lin-28 homolog B, Single nucleotide polymorphism

## Abstract

**Background:**

The present study aimed to investigate the association between MKRN3 and LIN28B gene polymorphisms and precocious puberty in Korean boys and girls.

**Results:**

Children 7 to 9 years of age in 2011 to 2012 who were part of the Ewha Birth & Growth Cohort Study were recruited for this study. A total of 103 girls and 70 boys were included in the analyses. Seven girls and 26 boys were identified to have precocious puberty. Among four single nucleotide polymorphisms (SNPs) of MKRN3 and two SNPs of LIN28B examined, three SNPs (rs2239669, rs6576457, and rs12441827) showed significant associations with precocious puberty in additive models in boys but no significance was found in any SNPs in girls. From the logistic regression analysis, boys with TT alleles in rs12441827 had about a four-times greater risk for precocious puberty when compared to C allele carriers (OR = 3.95, 95% CI = 1.27–12.32 in model 1). eQTL analysis revealed that SNPs of statistical significance from our study did not show the variation in expression profiles nor found in the database.

**Conclusions:**

This study supports the impact of MKRN3 SNP rs12441827 on precocious puberty in Korean boys. The results add a further aspect to genetic association in precocious puberty along with complex interactions of environmental, nutritional and socioeconomic factors.

## Background

Puberty is a complex developmental process required for reproductive competency. The onset of puberty is dependent on the activation of hypothalamus-pituitary-gonadal axis. Gonadotropin-releasing hormone (GnRH) plays a crucial role in reproduction and sexual development by leading to secretion of luteinizing hormone and follicle stimulating hormone, which in turn stimulates gonadal secretion of testosterone and estradiol [1]. Onset of puberty is regulated by complex interactions of environmental, genetic, nutritional, and socioeconomic factors [2, 3].

Precocious puberty is clinically defined by the development of secondary sexual characteristics before the age of 8 years in girls and 9 years in boys [4]. The prevalence of precocious puberty is substantially increasing in Western countries and Asia in both girls and boys [5]. The overall incidence of precocious puberty in Korea was 15.3 per 100,000 girls, and 0.6 per 100,000 boys between the years 2004 to 2010 [6]. Precocious puberty is associated with many problems including early bone maturation, reduced adult height, and increased risk for the development of psychological problems [7]. Pubertal timing varies within the population and differs between boys and girls [8]. There is evidence of the influence of genetic factors on pubertal timing [9–12]. Recently, genetic variation in and near the makorin RING finger protein 3 (MKRN3) was reported to be associated with cases of familial central precocious puberty as well as in non-familial central precocious puberty [13, 14]. Genome-wide association studies have identified several single nucleotide polymorphisms (SNPs) in or near the Lin-28 homolog B (LIN28B) gene as linked to age at menarche. Hence, it is possible that menarche related SNPs may be related to precocious puberty [15, 16].

Although the functions of these genes are not completely known, MKRN3 and LIN28B are regarded as important genes for the onset of puberty. However, few studies have been focused on these genes concerning precocious puberty in Korea and especially in boys. Therefore, we aimed to investigate the correlation between MKRN3 and LIN28B gene polymorphisms and precocious puberty in Korean girls and boys.

## Methods

### Subjects

As part of the Ewha Birth & Growth Cohort Study, we conducted the present investigation to assess the effects of gene polymorphisms on precocious puberty. The Ewha Birth & Growth Cohort Study involves a prospective birth cohort established in 2001–2006. A detailed explanation of this cohort has been provided in previous studies [17, 18]. Briefly, we recruited pregnant women at gestational age 24–28 weeks who sought prenatal care at the Ewha Womans University Mokdong Hospital, Seoul, Korea. A total of 940 women participated and their children have been followed-up since 2005. The physical exam was performed every year starting 2011 in girls 7 and 8 years old and in boy 7 to 9 years old. A total of 344 parents were telephoned and asked to participate in the study. Detailed follow-up data were obtained from July to August of 2011 and peripheral blood samples were collected in EDTA-coated tubes for genotype analysis during the follow-up visits [19]. The information collected included age, gender, height, body weight, and Tanner stage. Subjects were excluded if data on Tanner stage, height, or weight was missing. Breast and pubic hair development in girls and genital enlargement and pubic hair development in boys were evaluated by pediatrician in accordance with the Tanner stage system. Testes size was classified per Tanner staging, which stage 1 refers to testes smaller than 1.5 mL or long axis < 2.5 cm, stage 2 refers to size 1.6–6 mL or long axis 2.5–3.2 cm, and stage 3 refers to size 6–12 mL or long axis 3.3–4.0 cm [6].

This cohort study was performed in Ewha Womans University Mokdong Hospital. The study protocol was approved by ethics committee of the Ewha Womans University Mokdong Hospital Institutional Review Board, and written consent was obtained from the parents of each subject.

### Genotyping

Genomic DNA was obtained from peripheral blood collected at the follow-up visit. Genomic DNA was isolated from whole blood sample using the QIAamp DNA Blood Mini Kit (QIAGEN GmbH, Hilden, Germany) following the manufacturer’s protocol. Four SNPs of the MKRN3 gene (rs2239669, rs12441827, rs657457, and rs12148769) and two SNPs of the LIN28B gene (rs314280 and rs314276) were selected based on the data from previous studies [16, 20–22] and considering the minor allele frequency (MAF) of 20% or above in combined Chinese and Japanese (CHB + JPT) panels from Hapmap v3 (release #2). Primer sequences of these SNPs are provided in Table 1. The genotypes of five SNPs were screened using single base primer extension assay using ABI PRISM SNaPshot Multiplex kits (ABI, Foster City, CA, USA) according to manufacturer’s protocol. LIN28B rs314276 was analyzed by sequencing due to the failure of SNaPshot assay. Genotyping results have been deposited in the TreeBASE database (Study Accession URL: http://purl.org/phylo/treebase/phylows/study/TB2:S22535).

**Table 1:** Primer sequences used for genotyping study by SNaPShot method

Gene	rs number	Primer sequence	
MKRN3	rs2239669	F^a^	CCCTTTCCTTGCCTGTGAT
		R^b^	GGGGTTGGCCTTCTCATAG
		G^c^	GAGCTCTGTAGAGGAGCCTC
MKRN3	rs6576457	F	TTGCTTCATTCTGCTTTCATTG
		R	CCCATCTGTGCTGTGTTGTG
		G	GTATTCCTACTTTGCTGAGAGTA
MKRN3	rs12441827	F	TTGCTTCATTCTGCTTTCATTG
		R	CCCATCTGTGCTGTGTTGTG
		G	GTTTCTAATAAGAAGCCCTTCTGTC
MKRN3-MAGEL2 locus	rs12148769	F	CCCTGGTACAGGGTTTCCTT
		R	TCCAACTCTTGACCTCGTGA
		G	TGGAATTAAATTATGCATTGTCAA
LIN28B	rs314276	F	TGAATTAAAACATGTAGCTGCTGA
		R	TCGTCTTGAATTGCAACCTT
LIN28B	rs314280	F	TCTGGGGAAAGTGTGTTAAGG
		R	CGGGCAAACTGTCCTGAT
		G	GGAAACAARAGCAAAGCGACCCAACT

^a^Forward primer

^b^Reverse primer

^c^Genotyping primer

### Data analysis

Univariate analysis was conducted in order to identify the association of precocious puberty with factors including age, weight, height, and body mass index (BMI). Continuous variables were compared by the Independent-samples *t*-test. The *chi*-square test was done to compare categorical variables. Tests of normality were performed and determined by Shapiro-Wilk’s *p* value. Variables without normal distribution were analyzed by nonparametric method such as Mann-Whitney test. Each genotype of examined polymorphisms was assessed by unconditional logistic regression analyses under the additive (major allele homozygotes vs. heterozygotes or minor allele homozygotes vs. heterozygotes) models, dominant (major allele homozygotes vs. heterozygotes + minor allele homozygotes), and recessive (major allele homozygotes + heterozygotes vs. minor allele homozygotes) models, respectively. Pseudo-R-square statistics of Nagelkerke R Square were analyzed and Hosmer–Lemeshow test was used to assess the goodness of fit of multivariate model.

All statistical tests were two-sided and *p*-values < 0.05 were considered to be statistically significant. All analyses were performed with the Package for Social Sciences Version 23.0 for Windows (SPSS 23.0 K, SPSS INC, Chicago, IL, USA).

## Results

A total of 103 girls and 70 boys were included in the analyses. There were 96 girls (93.2%) in Tanner stage 1 at the age of 7 and 8 years, 7 girls in stage 2 at the age of 7 years (6.8%), and none in stage 3. Among boys, 44 (62.9%) were found to be in stage 1 at the age of 7 to 9 years, 21 (30.0%) in stage 2 at the age of 7 and 8 years, and 5 (7.1%) in stage 3 at the age of 7 and 8 years. Since Tanner stage 2 or above before the age of 8 years in girls and 9 years in boys represents precocious puberty, 7 girls and 26 boys were identified to have a precocious puberty. The mean age of the study participants was 7.3 years in girls and 7.9 years in boys. From univariate analysis, weight was a significant factor for the precocious puberty in girls but not in boys (Table 2).

**Table 2:** Baseline characteristics in association with precocious puberty

	Girls (*n* = 103)			Boys (*n* = 70)		
	Prepuberty	Precocious puberty	*P* value	Prepuberty	Precocious puberty	*P* value
Age, n (%)^a^			0.006			< 0.0001
7 years	61 (89.7)	7 (10.3)		11 (47.8)	12 (52.2)	
8 years	35 (100.0)	0 (0)		14 (50.0)	14 (50.0)	
9 years				19 (100.0)	0 (0)	
BMI (kg/m^2^)^b^	15.3 (14.7, 17.1)	16.0 (15.1, 19.1)	0.237	16.1 (15.2, 19.6)	15.8 (14.6, 18.2)	0.229
BMI z score^b^	−0.36 (−0.65, 0.48)	−0.07 (− 0.49, 1.37)	0.237	−0.33 (− 0.66, 0.97)	−0.42 (− 0.88, 0.52)	0.308
Weight (kg)^b^	24.3 (22.2, 27.6)	26.7 (25.8, 30.9)	0.057	27.5 (24.2, 35.1)	25.9 (22.7, 31.6)	0.073
Height (cm)	124.7 (6.2)	127.6 (3.4)	0.217	130.8 (8.5)	128.6 (6.4)	0.273
Height SDS	−0.03 (1.0)	0.45 (0.6)	0.217	0.10 (1.1)	−0.17 (0.8)	0.273
Tanner stage^a^						
I	96 (93.2)			44 (62.9)		
II		7 (6.8)			21 (30.0)	
III		0 (0.0)			5 (7.1)	
Testes						< 0.0001
I				44 (74.6)	15 (25.4)	
II				0 (0)	10 (100.0)	
III				0 (0)	1 (100.0)	

Abbreviation: *BMI* body mass index, *SDS* standard deviation score

^a^The variables are given in mean (standard deviation)

^b^Data shown in median (interquartile range)

Haploview analyses indicated high linkage disequilibrium (LD) between rs2239669 and rs6576457 (r^2^ = 0.97 in girls and r^2^ = 1 in boys) and rs314280 and rs314276 (r^2^ = 0.97 in girls and r^2^ = 0.96 in boys) in this population.

Additive model analysis revealed significant associations between three SNPs in MKRN3 (rs2239669, rs6576457, and rs12441827) and precocious puberty in boys (OR = 7.73, 9.27, and 0.17, 95% CI = 1.54–38.67, 2.89–45.48, and 0.08–0.37, respectively). Also, rs12441827 showed statistical significance in dominant models (OR = 0.18, 95% CI = 0.06–0.51) and rs2239669 and rs6576457 were in significant association with precocious puberty in recessive models (OR = 10.75 and 12.9, 95% CI = 1.78–98.15 and 1.46–114.40, respectively) in boys. In contrast, none of the SNPs showed statistical significance with precocious puberty in girls. Also, SNPs in the LIN28B gene were not associated with precocious puberty in both boys and girls (Table 3).

**Table 3:** Genotypes of 6 SNPs

Genotype	Girls		Boys	
	Prepuberty (%)	Precocoious puberty (%)	Prepuberty (%)	Precocious puberty (%)
rs2239669^a,b^				
CC	58 (62.4)	4 (57.1)	26 (59.1)	9 (36.0)
CT	28 (30.1)	2 (28.6)	17 (38.6)	11 (44.0)
TT	7 (7.5)	1 (14.3)	1 (2.3)	5 (20.0)
rs6576457^a,c^				
GG	59 (62.8)	4 (57.1)	26 (59.1)	9 (34.6)
GA	26 (27.7)	2 (28.6)	17 (38.6)	11 (42.3)
AA	9 (9.6)	1 (14.3)	1 (2.3)	6 (23.1)
rs12441827^d,e^				
TT	44 (45.80)	2 (28.6)	11 (25.0)	17 (65.4)
TC	38 (39.6)	5 (71.4)	30 (68.2)	8 (30.8)
CC	14 (14.6)	0 (0)	3 (6.8)	1 (3.8)
rs12148769				
GG	51 (53.1)	3 (42.9)	21 (47.7)	15 (57.7)
GA	36 (37.5)	3 (42.9)	20 (45.5)	11 (42.3)
AA	9 (9.4)	1 (14.3)	3 (6.8)	0 (0)
rs314276				
CC	55 (57.3)	3 (42.9)	27 (61.4)	16 (61.5)
CA	36 (37.5)	3 (42.9)	15 (34.1)	7 (26.9)
AA	5 (5.2)	1 (14.3)	2 (4.5)	3 (11.5)
rs314280				
AA	5 (5.3)	1 (14.3)	2 (4.5)	3 (11.5)
GA	35 (36.8)	3 (42.9)	14 (31.8)	7 (26.9)
GG	55 (57.9)	3 (42.9)	28 (63.6)	16 (61.5)

^a^*P* < 0.01 for additive model (minor allele homozygotes versus heterozygotes) in boys

^b^*P* < 0.05 for recessive model in boys

^c^*P* < 0.01 for recessive model in boys

^d^*P* < 0.0001 for additive model (major allele homozygotes versus heterozygotes) in boys

^e^*P* < 0.01 for dominant model in boys

Multivariate stepwise regression model was used to clarify independent association between three variants ((rs2239669, rs6576457, and rs12441827) and precocious puberty in boys. The results confirmed that rs12441827 SNP was strong independent factor associated with precocious puberty. This indicated boys with TT alleles in rs12441827 have about four-times greater risk for precocious puberty when compared to boys carrying C allele (OR = 3.95, 95% CI = 1.27–12.32 in model 1) (Table 4).

**Table 4:** Multivariate logistic regression in boys

Variables	Model 1		Model 2		Model 3	
	Adjusted odds ratio (95% CI)	*P* value	Adjusted odds ratio (95% CI)	*P* value	Adjusted odds ratio (95% CI)	*P* value
Body mass index (kg/m^2^)	0.90 (0.72–1.12)	0.349				
rs2239669		0.182				
TT	0.21 (0.02–2.10)		0.22 (0.22–2.22)	0.199		
CT, CC	1		1			
rs12441827		0.018		0.016		0.002
TT	3.95 (1.27–12.32)		4.03 (1.30–12.48)		5.33 (1.84–15.46)	
TC, CC	1		1		1	

The odds ratio was adjusted for body mass index, rs12441827, rs2239669, and rs6576457

Hosmer-Lemeshow goodness-of-fit test, *χ*^2^ = 9.877, *p* = 0.274

Nagelkerke’s R^2^ in model 1 = 0.238, model 2 = 0.223, model 3 = 0.188

In order to understand the effects of chosen SNPs on MKRN3 gene expression, we examined the expression profiling using GTEx datasets [23]. eQTL analysis was performed on Genotype-Tissue Expression (GTEx) dataset which contains RNA sequencing and whole genome sequencing data from non-diseased human tissues across 960 donors [24]. Unfortunately, three SNPs (rs12441827, rs6576457, rs2239669) were not found in GTEx datasets. The SNP rs12441827 was recorded as a significant expression quantitative trait loci (eQTL) with MKRN3 transcript (*p* = 5.1 × 10^− 7^), although we could not find a significant difference of the chosen genotype frequency with precocious puberty in our cohort. The expression profiles for LIN28B revealed significant regional expression differences showing lower expression with variant type alleles in rs314276 and wild type alleles in rs314280 in pituitary (*p* = 9.4 × 10^− 2^ and *p* = 7.6 × 10^− 8^, respectively). However, both SNPs of LIN28B were not shown to be associated with precocious puberty in our study.

## Discussion

This study aimed to investigate the association between MKRN3 and LIN28B gene polymorphisms and precocious puberty in Korean children by analyzing six SNPs. From the multivariate regression analysis, a significant association between rs12441827 mutation within the MKRN3 gene and a decreased risk for precocious puberty in boys was observed.

MKRN3 is an intronless gene located on chromosome 15q11.2, in the Prader–Willi syndrome critical region [25]. The role of MKRN3 in pubertal initiation was first described in a genetic study of several families with precocious puberty in 2013. In this study, the authors researched 40 members of 15 families with precocious puberty from several ethnic groups (12 Brazilian families, 2 American families, and one Belgian familys) and demonstrated the effect of mutations of MKRN3 for precocious puberty. Settas et al. described a novel heterozygous missense mutation (p.Cys340Gly) in MKRN3 in two Greek siblings, a girl with central precocious puberty and a boy with early puberty [26]. These studies further expanded the MKRN3 mutational spectrum in familial precocious puberty on top of the findings that MKRN3 gene is expressed from paternally inherited copy [27].

We studied MKRN3 polymorphisms in unrelated children (103 girls and 70 boys) and identified an association with precocious puberty in boys but not in girls. This could be due to the lower incidence rate of precocious puberty in girls in our cohort compared to previous studies reporting higher frequency in girls than in boys [6, 28, 29]. Another reason that we could not find an association with SNPs in MKRN3 and precocious puberty in girls could be related to the lack of information on family history. MKRN3 is a paternally expressed genes but we were not able to consider whether the SNP was inherited from the father or not.

Although the function of MKRN3 gene is not completely understood, it is known to lead the inhibition of factors promoting the pulsatile GnRH secretion [30]. MKRN3 expression of male and female mice are comparable in hypothalamic arcuate nucleus which indicates prepubertal inhibitory tonus of MKRN3 in both sexes, but allows the possibility of gender-specific action [1, 31]. Girls begin puberty at a younger age than boys and are linked to higher rate of central precocious puberty while boys have a higher incidence of delayed puberty [32]. It’s likely to be indicated that the inhibition of GnRH during childhood is weaker in girls than in boys allowing girls to be more sensitive to disturbances in pubertal onset.

The association of MKRN3 polymorphism and precocious puberty was studied previously and the author reported low frequency of MKRN3 mutations in central precocious puberty in Korean girls [33]. Bessa et al. demonstrated a high frequency of MKRN3 mutations in boys when compared to previously reported female data (40% versus 6.4%, respectively, *p* < 0.001), which is consistent with our findings [34]. The observed MAF of rs2239669 was 0.16 in a study done in Italian girls with idiopathic CPP, which is lower than the reported MAF of European population [35]. Another study done in France reported higher frequency of mutations in MKRN3 coding region [13]. These results show MKRN3 mutations are common in familial idiopathic CPP and lower in sporadic cases. Although our study included small number of children, the result is meaningful as this is the first study to investigate the association of MKRN3 and precocious puberty in boys in Korea. Also, we described novel finding that rs12441827 is associated with precocious puberty, which is the first report regarding this SNP.

We analyzed two SNPs (rs314276 and rs314280) in LIN28B and found no significant association with precocious puberty in both girls and boys in all models. Some studies reported rs314280 and rs314276 were significantly associated with precocious puberty [15, 16] while others failed to show significance in those SNPs [36, 37]. This inconsistency could be explained by the small sample size or the ethnic differences. Certainly, as other SNPs within LIN28B need to be investigated including non-coding regions. Given the finding of a study in mice that genetic modification of the Lin28-Let-7 pathway by over-expression of Lin28B increased body size and delayed the onset of puberty [38], involvement of LIN28B in the regulation of pubertal timing in human still warrants further studies in different patient groups and populations.

One of the limitation of the present study is the low statistical power due to the small number of samples, as calculated adequate sample size was 379 in each group [6, 13]. This warrants further investigation with larger sample size. Another limitation is that GnRH stimulation test was not performed. The diagnosis of precocious puberty was done by pediatrician through physical exams including breast development, size of the testes and penis, examination of pubic hair. Furthermore, for a comprehensive relationship between SNPs and precocious puberty, SNPs in coding region also needed to be analyzed in a near future.

## Conclusion

In conclusion, our study has found evidence that rs12441827 of MKRN3 is associated with precocious puberty in Korean boys. The exact mechanisms involved in pubertal development are still not well understood. Therefore, functional researches and studies with large-scale samples with MKRN3 and IN28B mutations are warranted to confirm our findings.

## Abbreviations

BMI: Body mass index
eQTL: Expression quantitative trait loci
GnRH: Gonadotropin-releasing hormone
LD: Linkage disequilibrium
LIN28B: Lin-28 homolog B
MAF: Minor allele frequency
MKRN3: Makorin RING finger protein 3
SNP: Single nucleotide polymorphism
